# Deep learning-enhanced zero echo time MRI for glenohumeral assessment in shoulder instability: a comparative study with CT

**DOI:** 10.1007/s00256-024-04830-0

**Published:** 2024-11-22

**Authors:** Laura Carretero-Gómez, Maggie Fung, Florian Wiesinger, Michael Carl, Graeme McKinnon, José de Arcos, Sagar Mandava, Santiago Arauz, Eugenia Sánchez-Lacalle, Satish Nagrani, Juan Manuel López-Alcorocho, Elena Rodríguez-Íñigo, Norberto Malpica, Mario Padrón

**Affiliations:** 1 GE HealthCare, Munich, Germany; 2 Medical Image Analysis and Biometry Lab, Rey Juan Carlos University, Madrid, Spain; 3 GE HealthCare, New York, NY USA; 4 GE HealthCare, San Diego, CA USA; 5 GE HealthCare, Waukesha, WI USA; 6 GE HealthCare, Little Chalfont, Amersham, UK; 7 GE HealthCare, Atlanta, GA USA; 8 Shoulder Unit, Clínica CEMTRO, Madrid, Spain; 9 Department of Radiology, Clínica CEMTRO, Madrid, Spain; 10 Research Unit, Clínica CEMTRO, Madrid, Spain

**Keywords:** Zero echo time, Deep learning, CT-like MRI, Shoulder, Instability, Bipolar bone loss

## Abstract

**Purpose:**

To evaluate image quality and lesion conspicuity of zero echo time (ZTE) MRI reconstructed with deep learning (DL)-based algorithm versus conventional reconstruction and to assess DL ZTE performance against CT for bone loss measurements in shoulder instability.

**Methods:**

Forty-four patients (9 females; 33.5 ± 15.65 years) with symptomatic anterior glenohumeral instability and no previous shoulder surgery underwent ZTE MRI and CT on the same day. ZTE images were reconstructed with conventional and DL methods and post-processed for CT-like contrast. Two musculoskeletal radiologists, blinded to the reconstruction method, independently evaluated 20 randomized MR ZTE datasets with and without DL-enhancement for perceived signal-to-noise ratio, resolution, and lesion conspicuity at humerus and glenoid using a 4-point Likert scale. Inter-reader reliability was assessed using weighted Cohen’s kappa (*K*). An ordinal logistic regression model analyzed Likert scores, with the reconstruction method (DL-enhanced vs. conventional) as the predictor. Glenoid track (GT) and Hill-Sachs interval (HSI) measurements were performed by another radiologist on both DL ZTE and CT datasets. Intermodal agreement was assessed through intraclass correlation coefficients (ICCs) and Bland–Altman analysis.

**Results:**

DL ZTE MR bone images scored higher than conventional ZTE across all items, with significantly improved perceived resolution (odds ratio (OR) = 7.67, *p* = 0.01) and glenoid lesion conspicuity (OR = 25.12, *p* = 0.01), with substantial inter-rater agreement (*K* = 0.61 (0.38–0.83) to 0.77 (0.58–0.95)). Inter-modality assessment showed almost perfect agreement between DL ZTE MR and CT for all bone measurements (overall ICC = 0.99 (0.97–0.99)), with mean differences of 0.08 (− 0.80 to 0.96) mm for GT and − 0.07 (− 1.24 to 1.10) mm for HSI.

**Conclusion:**

DL-based reconstruction enhances ZTE MRI quality for glenohumeral assessment, offering osseous evaluation and quantification equivalent to gold-standard CT, potentially simplifying preoperative workflow, and reducing CT radiation exposure.

## Introduction

The shoulder joint is the most frequently dislocated joint [1, 2], with approximately 1–2% of the general population experiencing a glenohumeral dislocation in their lifetime [3]. Over 95% of these events occur in the anterior direction, predominantly affecting young, active individuals [4, 5].

**Fig. 1:**
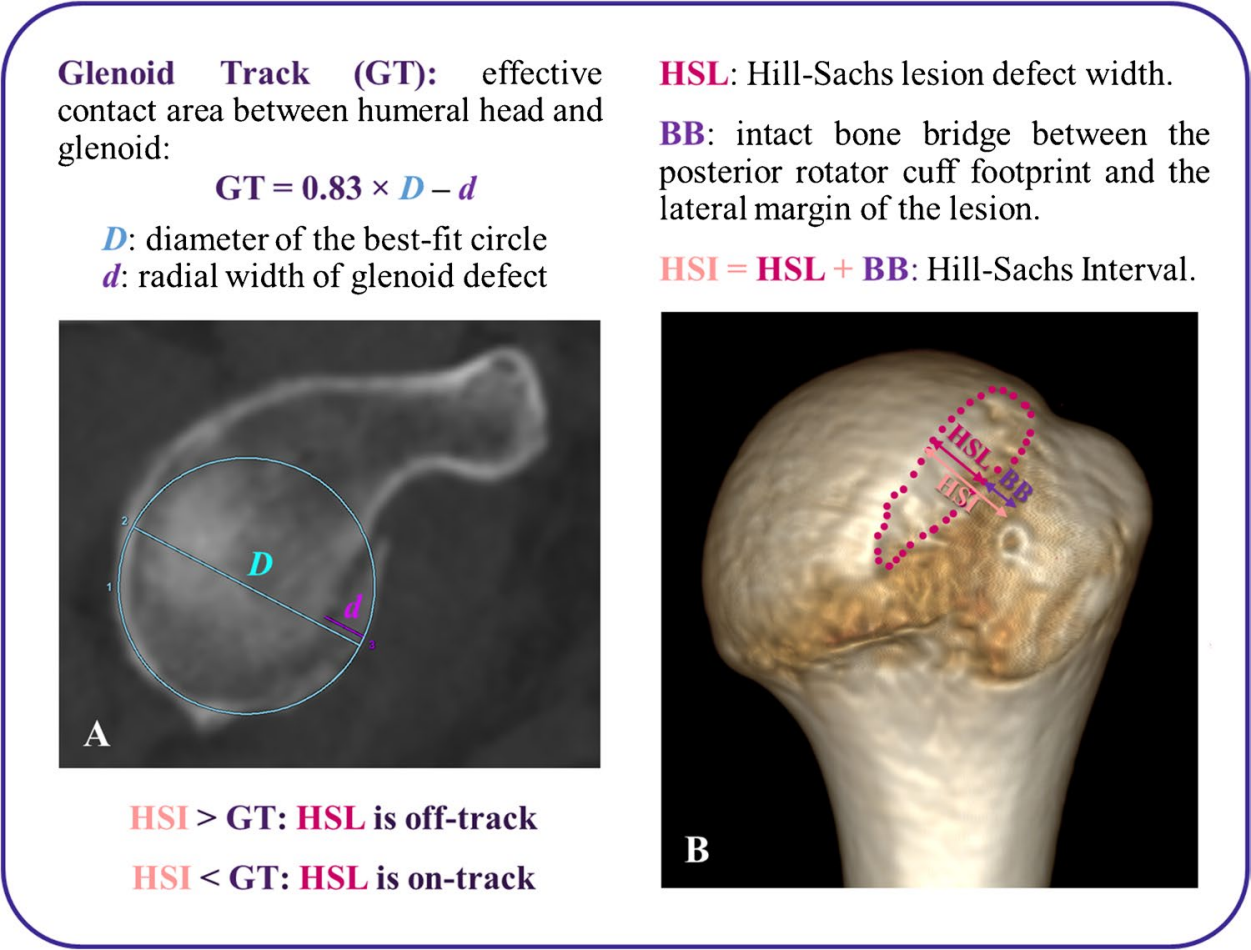
**Bipolar bone loss.** When the anterior glenoid rim is fractured, the GT is decreased as indicated in the equation: GT = 0.83 × *D* − *d*, where *D* is the glenoid joint face diameter and *d* is the glenoid rim defect (**A**). Hill-Sachs lesion is delimited by a dotted pink line. To properly account for the whole Hill-Sachs interval, the intact bone bridge must be added (**B**). The relationship between the GT and the HSI determines if a lesion is engaging (off-track) or stable (on-track)

Osseous defects in the glenoid and humeral head significantly increase instability risk. Hill-Sachs lesions (HSL) in the proximal humerus occur in over 67% of anterior shoulder dislocations and nearly 100% of patients with recurrent instability [6–9]. Bone loss exceeding 25% of the glenoid surface is critical for the recurrence of instability [10–13]. Consideration of the interactions of this bipolar bone loss has been used to determine the risk of engagement and select the appropriate surgical technique [14, 15]. The glenoid track (GT) is the contact zone of the glenoid on the humeral head during shoulder movements [11, 16]. In this article, we use the most recent value of 83% obtained in in vivo shoulders, as the effective track on an intact glenoid joint face diameter [17], diminishing with glenoid bone loss as shown in Fig. 1A. For the humerus side, there is typically an intact bone bridge (BB) between the rotator cuff attachments and the lateral margin of the HSL. The sum of the largest width (short axis) of the HSL and the width of the intact BB determines the Hill-Sachs interval (HSI) (Fig. 1B). When the medial rim of the HSI extends beyond the glenoid rim, there is an off-track unstable engaging lesion [11, 16].

Successful long-term outcomes can be compromised by failure to quantify bone loss and assess the risk of engagement [8, 18, 19]. Hence, the clinical workup to identify general risk factors (age, sex, ligamentous laxity, sporting activities, etc.) must be followed by a careful imaging assessment. Although 3D CT is the gold standard for bone assessment and presurgical planning due to its high correlation with arthroscopic evaluation [20, 21], MRI remains the imaging modality of choice for soft tissue evaluation, which is also key for operative management [6, 7]. The combined assessment of soft tissue and bone in patients with shoulder instability in a single one-stop MR examination would be highly desirable. Especially since shoulder instability often occurs in an active younger cohort, it would be preferable to avoid CT radiation in the chest/shoulder area if MRI-based bone imaging can provide similar information as CT.

While conventional MRI sequences with echo times (TE) in the millisecond range are generally too insensitive for meaningful bone signal detection due to their low proton density (~ 20% of water) and short signal lifetimes (T2 ~ 0.390 ms at 3 T [T]), resulting in cortical bone displaying as void at the time of acquisition, the 3D zero echo time (ZTE) technique has emerged as a valuable diagnostic tool in osteoarticular MRI. This technique acquires signals from short T2 tissues and can depict cortical bone surfaces unlike other MRI techniques [22, 23]. It has been adapted to image the osseous structures of the shoulder [24, 25], as well as the head [26, 27] and pelvis [28], and is increasingly being used in a wide range of musculoskeletal diseases, including osteoarthritis [29], degenerative diseases [30], tumors [31], and trauma [32].

Despite 3D imaging encoding, standard ZTE techniques are prone to low signal-to-noise ratio (SNR) and resolution compared to CT [33, 34]. Recently, introduced deep learning (DL)-based image reconstruction methods have demonstrated significant improvements in image quality (i.e., SNR and sharpness) and/or image encoding efficiency (i.e., scan time, coverage) [35].

The purpose of this study was to assess the image quality and lesion conspicuity of ZTE MRI reconstructed with a DL-based algorithm versus conventional reconstruction and to evaluate the quantitative accuracy of DL ZTE against gold-standard CT for bone loss measurements in patients with shoulder instability. We hypothesized that applying an investigational DL reconstruction model to the standard ZTE technique would increase diagnostic confidence, offering similar results to CT, while avoiding radiation and improving patient workflow with a single MR examination.

## Methods

### Patient selection

After institutional review board approval and written informed consent, 44 patients were recruited for this cohort study. Patients were referred by orthopedic surgeons highly specialized in shoulder management and sports medicine, from our institution’s daily schedule between June 2021 and June 2022.

Inclusion criteria were clinically symptomatic anterior glenohumeral instability and recurrent dislocations. CT and MRI examinations were scheduled consecutively on the same day. Patients who had contraindications to MRI or previous shoulder surgery were excluded.

### Imaging protocol

CT scanning was performed on a 256-slice Revolution™ CT scanner (GE HealthCare, Chicago, IL) with the following scan parameters: slice thickness, 1.25 mm; reconstruction matrix, 512 × 512 pixels; field of view, 20 to 30 cm; 140 kV (peak); and pitch factor 0.98.

MRI was conducted on a 3 T SIGNA™ Architect MR scanner (GE HealthCare, Chicago, IL) with a 20-channel AIR™ multi-purpose coil, adding to the routine shoulder clinical protocol a 3D radial ZTE sequence [24]; flip angle (FA) = 1°, repetition time (TR) = 88.6 ms, isotropic field of view (FOV) = (180 mm)^3^, isotropic resolution = (1.0 mm)^3^, bandwidth = ± 62.5 kHz, number of averages (NEX) = 4, acquisition time = ~ 2 min. This protocol takes advantage of DL-enhanced reconstruction, by lowering NEX (and hence the scan time) to half the value conventionally used (i.e., NEX = 6–8 with 3–4-min scan time) [25]. The patient positioning was consistent across modalities, with the head-first supine orientation and the shoulder joint positioned as close as possible to the center of the table.

**Fig. 2:**
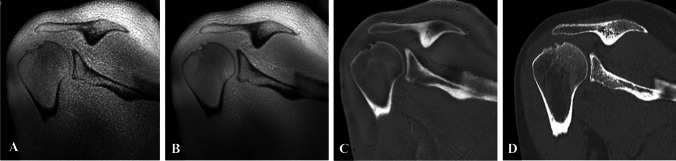
**Oblique coronal images illustrating the post-processing of ZTE data for CT-like positive bone contrast in one of the subjects. **Native ZTE MR image (**A**). ZTE after DL recon (**B**). MR ZTE bone image–bias-corrected DL ZTE followed by signal inversion and background suppression (**C**). CT reference image (**D**)

The DL reconstruction method employed to improve SNR and sharpness of ZTE MRI is an extension of the commercially available AIR™ Recon DL deep learning-based reconstruction pipeline (GE HealthCare, Chicago, IL) [35]. This algorithm uses a deep convolutional neural network operating on raw k-space data and has been trained to reconstruct images with minimal noise and ringing artifacts. Once ZTE images were reconstructed with both conventional and DL methods, a bias-correction algorithm was applied to correct for signal inhomogeneities due to coil geometry, followed by inverse linear scaling and bright background air suppression to provide CT-like contrast (Fig. 2). These post-processing steps were run automatically on the reconstructed ZTE DICOM images.

### Radiological assessment

Two radiologists blinded to the ZTE reconstruction method (i.e., conventional vs DL), each with more than 15 years of experience in musculoskeletal imaging, independently evaluated 20 randomized ZTE MR bone datasets (i.e., ZTE and DL ZTE) for perceived SNR, perceived resolution, and lesion conspicuity (cortical bone depiction) at both humerus and glenoid sides, using a 4-point Likert scale (1 = non-diagnostic, 2 = poor, 3 = adequate, 4 = excellent).

### Osseous measurements

Another musculoskeletal radiologist (5 years of experience) blinded to the patient’s history evaluated for bipolar bone loss in both DL ZTE and CT datasets independently, by measuring the GT and the HSI on a GE AW workstation (GE HealthCare, Chicago, IL).

**Fig. 3:**
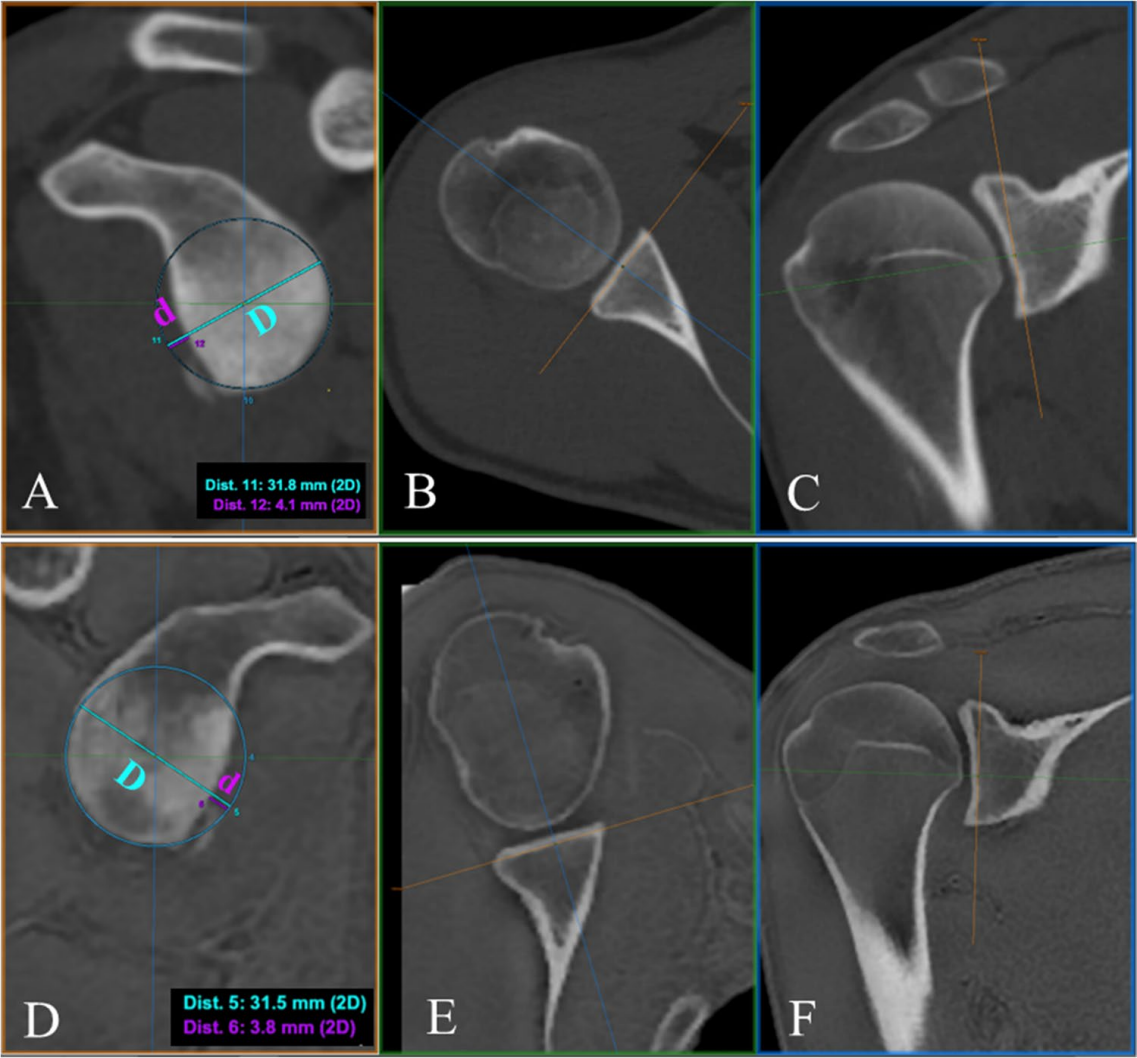
**GT measurement.** En face sagittal MPR view of the glenoid showing the best-fit circle placement on CT (**A**) and ZTE (**D**) images from the same patient, with proper angulation on their corresponding axial (**B**, **E**) and coronal (**C**, **F**) planes

GT was quantified on a 2D reformatted sagittal oblique “en face” view of the glenoid making use of the multiplanar reformation (MPR) [36] and the best-fit circle technique [37]. The angulation of the sagittal oblique axis (orange lines in Fig. 3) on both coronal and axial planes was done according to the inclination of the lower two-thirds of the glenoid joint face and not according to the glenoid inclination, since this may lead to erroneous representation of the glenoid rim defect. Once the sagittal oblique plane was determined, the circle was placed fitting the inferior two-thirds of the pear-shaped glenoid cavity and aligned with the posteroinferior margin (Fig. 3A, D), measuring the corresponding diameter (*D*). In those cases that presented glenoid bone loss, the maximum radial width of defect (*d*) was measured as the distance between the remnant margin of the anterior glenoid and the anterior margin of the circle. The width of the GT was computed by the formula shown in Fig. 1.

Additionally, volume renderings (VR) of the en face glenoid by removing the humeral head were also obtained in two cases to compare them with the 3D CT VR.

HSI was measured on the 3D VR where all the bone components except for the humeral head were cut off to improve its segmentation. MPR was also used to cross-reference with the 2D planes and help to locate the rims of the HSI.

**Fig. 4:**
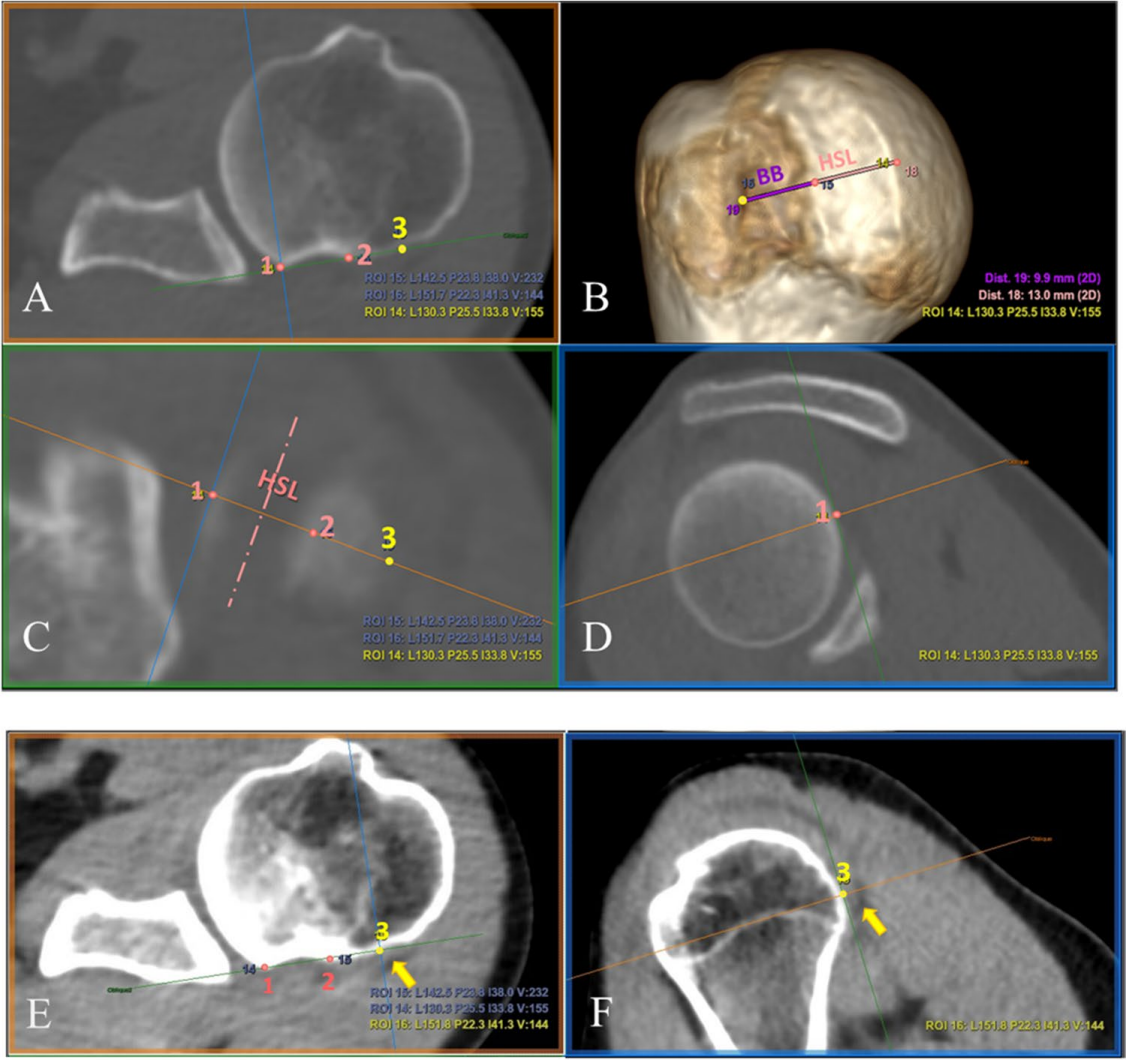
**HSI** **measurement on CT**. Points 1 and 2 (**A**) determine the limits of the HSL, which is perpendicular to the longitudinal axis of the HSI (represented by the pink dashed line (**C**)). (**D**) Sagittal view showing the medial most extent of the lesion (point 1). Point 3 in yellow represents the rotator cuff footprint, which is determined by forcing soft tissue windowing, where the tendon insertion is seen with higher intensity (yellow arrow (**E**, **F**)). The distances between the reference points are measured on 3D VR (**B**)

**Fig. 5:**
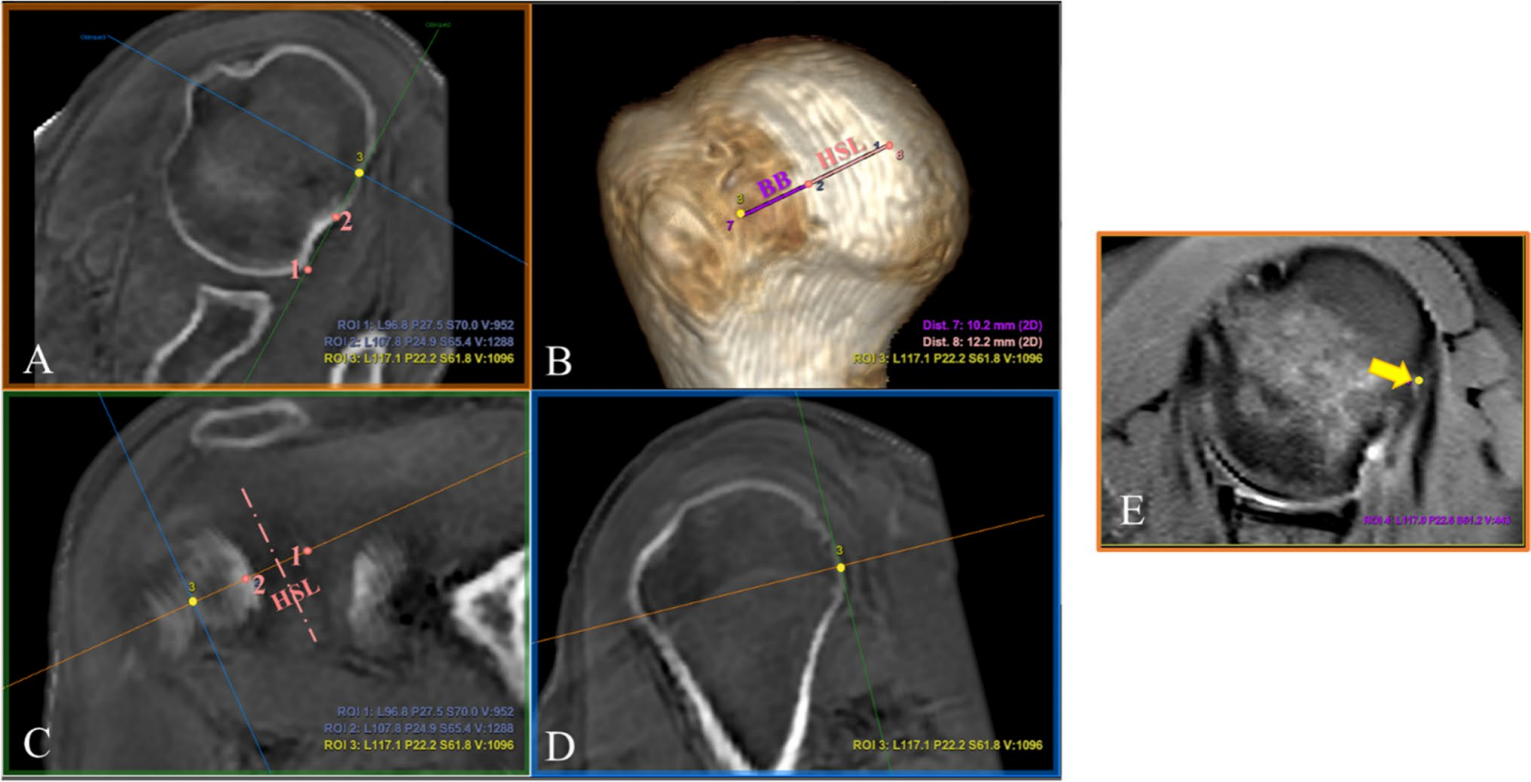
**HSI measurement on DL ZTE.** Points 1 and 2 determine the limits of the HSL (**A**), which is perpendicular to the longitudinal axis of the HSI (represented by the pink dashed line (**C**)). Point 3 in yellow represents the rotator cuff footprint (**D**), which is cross-referenced with the propeller routine series (**E**) to confirm the tendon insertion (yellow arrow). On the 3D VR (**B**), the distances between the reference points are measured

A recently published MPR methodology provides a standardized way to find out the reference points of the HSI [38]. By scrolling through the slices on the axial plane, the medial most extent of the HSL can be identified (point 1 in Fig. 4). By properly rotating the axial plane (orange line) in both coronal (C) and sagittal (D) views, it was possible to identify the longitudinal axis of the HSL (pink dashed line in (C)), and consequently rotate and place the short axis of the HSL perpendicular to it. The lateral extent of the HSL (point 2) can be placed along this identified short axis on the coronal view (C). The resulting axial image (A) that corresponds with the oblique plane passing through the short axis is used to identify the medial edge of the rotator cuff footprint (tendon insertion, point 3) and determine whether there is a BB. On DL ZTE, this is facilitated by registering and cross-referencing with the inherent soft tissue contrast enhancement provided by the routine MR images (Fig. 5E) whereas on CT soft tissue windowing must be forced to outline the tendon attachment (Fig. 4E, F).

### Statistical analysis

Frequencies and percentages were used to report radiologists’ Likert scores. Inter-reader reliability was assessed using weighted Cohen’s kappa (*K*) using four-level ordinal weights. An ordinal logistic regression was used to assess the effect of reconstruction method (conventional vs. DL) on the Likert scores for each feature. Odds ratios (ORs) with their 95% CI were reported.

Intraclass correlation coefficient (ICC) values were calculated to assess intermodal agreement on measuring GT and HSI. Bias and limit of agreement (LoA) values (1.96 × standard deviation of difference) were calculated with the methods of Bland and Altman [39].

Statistical significance level was established a priori as *p* < 0.05. Agreement interpretation was also selected a priori according to published standards [40]: < 0.01, poor; 0.01–0.20, slight; 0.21–0.40, fair; 0.41–0.60, moderate; 0.61–0.80, substantial; and 0.81–1.0, almost perfect agreement. All statistics were performed using GraphPad Prism (GraphPad Software, Boston, MA, USA).

## Results

The study cohort consisted of 44 patients with clinically symptomatic anterior shoulder instability (9 females and 35 males; mean ± SD age, 33.5 ± 15.65 years; range, 16–76 years). Mean age of first dislocation was 28 years. Twenty-six patients had 3 or less total shoulder dislocations prior to consultation, 10 had between 4 and 10, and 8 had more than 10. Median time from the first dislocation to the consultation was 2 years (IQR 0–10 years). Twenty-one patients presented bipolar bone loss whereas 23 only Hill-Sachs lesions.

### Radiological assessment

DL ZTE images were more often rated as adequate or excellent compared to conventional ZTE (Table 1). There was substantial inter-rater agreement for all the items assessed: perceived SNR (0.76 (0.57–0.95)), perceived resolution (0.61 (0.38–0.83)), and lesion conspicuity at both humerus (0.65 (0.41–0.88)) and glenoid regions (0.77 (0.58–0.95)).

**Table 1:** **Radiological assessment using Likert scores (2 = poor, 3 = adequate, 4 = excellent)**. No cases received a non-diagnostic score (1). Data are presented as frequency (percentage) for each score

Item	Likert score	Reader 1		Reader 2	
		Conventional	DL	Conventional	DL
Perceived SNR	2	12 (60%)	0 (0%)	16 (80%)	0 (0%)
	3	8 (40%)	2 (10%)	4 (20%)	5 (25%)
	4	0 (0%)	18 (90%)	0 (0%)	15 (75%)
Perceived resolution	2	6 (30%)	0 (0%)	8 (40%)	0 (0%)
	3	14 (70%)	6 (30%)	12 (60%)	4 (20%)
	4	0 (0%)	14 (70%)	0 (0%)	16 (80%)
HSL conspicuity	2	1 (5%)	0 (0%)	0 (0%)	0 (0%)
	3	11 (55%)	2 (10%)	16 (80%)	0 (0%)
	4	8 (40%)	18 (90%)	4 (20%)	20 (100%)
Glenoid conspicuity	2	12 (60%)	0 (0%)	11 (55%)	0 (0%)
	3	6 (30%)	7 (35%)	9 (45%)	8 (40%)
	4	2 (10%)	13 (65%)	0 (0%)	12 (60%)

For perceived SNR, the odds of receiving a higher score with DL-enhanced ZTE were not statistically significant, with an OR of 3.00 (0.32–28.10, *p* = 0.34). In contrast, DL-enhanced ZTE significantly increased the odds of higher perceived resolution scores compared to conventional ZTE, with an OR of 7.67 (1.56–37.80, *p* = 0.01). The analysis for Hill-Sachs lesion conspicuity revealed a positive but non-significant effect, with an OR of 4.58 (0.12–168.57, *p* = 0.41). Finally, DL-enhanced ZTE had a significant impact on glenoid lesion conspicuity, increasing the odds of a higher score by 25.12 times compared to conventional ZTE (95% CI, 2.82–223.54; *p* = 0.01).

### Inter-modality agreement

**Fig. 6:**
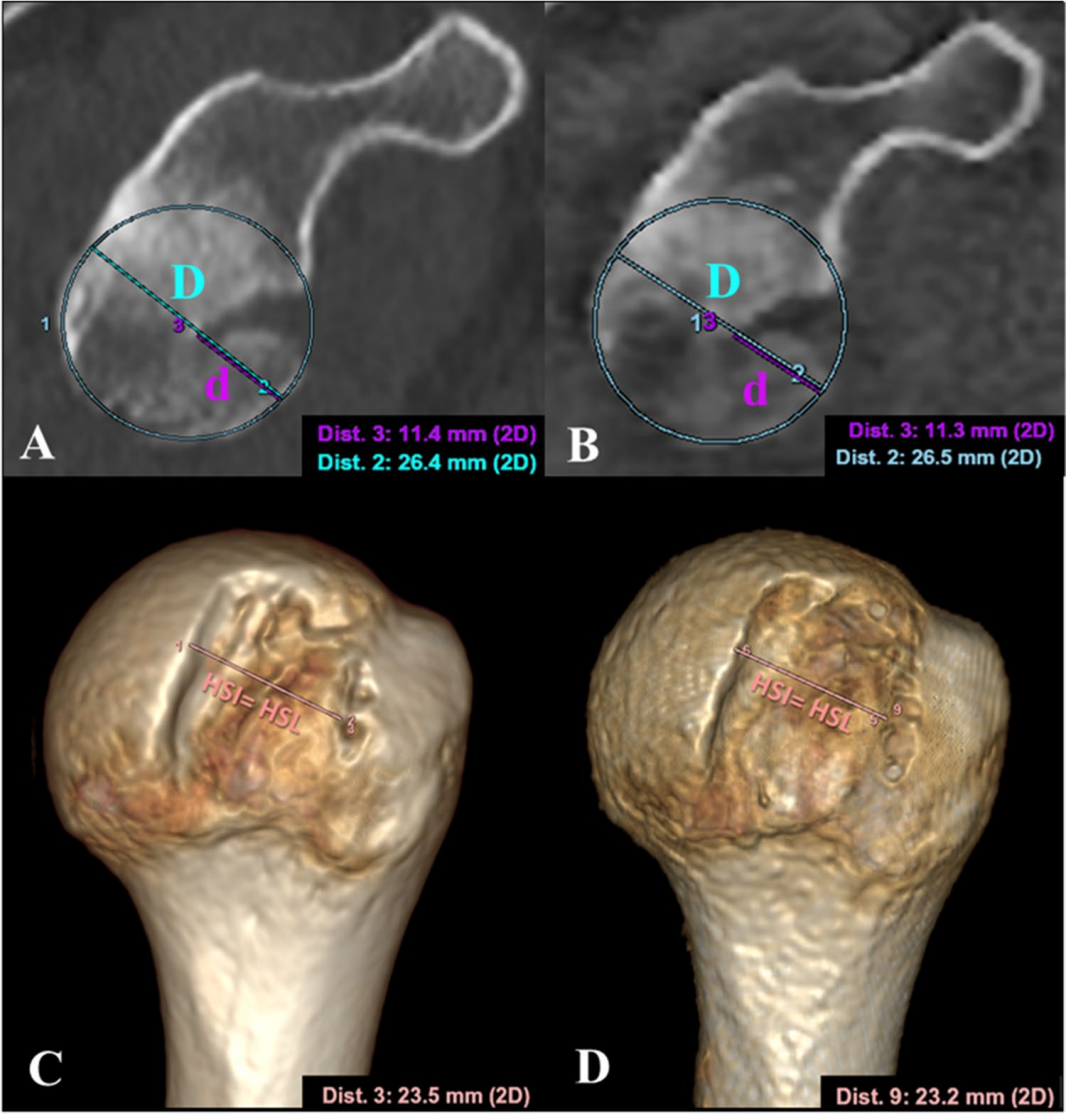
**Bipolar bone loss measurement.** GT measurement through the best-fit circle on sagittal en face glenoid view on CT (**A**) and bone image from DL ZTE (**B**). Both diameter (*D*) and glenoid bone loss (*d*) are shown in blue and purple respectively. Leading to a GT of 10.51 mm for CT and 10.70 mm for DL ZTE. HSI measurement on 3D volumes, CT (**C**) and DL ZTE (**D**). In this case, there is no BB, so all the HSI corresponds to the HSL, 23.5 mm for CT (**C**) and 23.2 mm for ZTE (**D**)

The reader found excellent concordance for measurements obtained from both modalities (Fig. 6). ICCs (95% CI, *p* < 0.0001) for inter-modality assessment showed almost perfect agreement for both measurements: GT = 0.99 (0.98, 0.99) and HSI = 0.99 (0.97, 0.99).

**Fig. 7:**
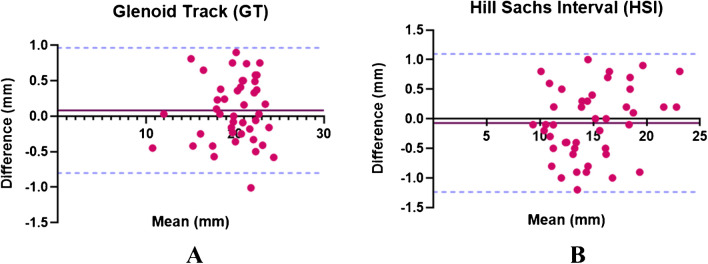
**Bland–Altman plots of 3D CT–3D DL ZTE for glenoid track** (**A**) **and Hill-Sachs interval** (**B**) **measurements**. The solid lines represent the mean differences (bias), and the dashed lines indicate the 95% limits of agreement. It is expected that the limits include 95% of differences between the 2 measurement methods. Each study participant is represented by an individual point

Bland–Altman analysis revealed a bias (mean difference) of 0.08 (− 0.80 to 0.96) mm for GT and − 0.07 (− 1.24 to 1.10) mm for HSI measurements using both techniques. Bland–Altman plots of agreement demonstrated no discernable pattern to the distribution of differences between measurements over the range of measured values (Fig. 7), showing a high inter-modality agreement between CT and DL ZTE.

**Fig. 8:**
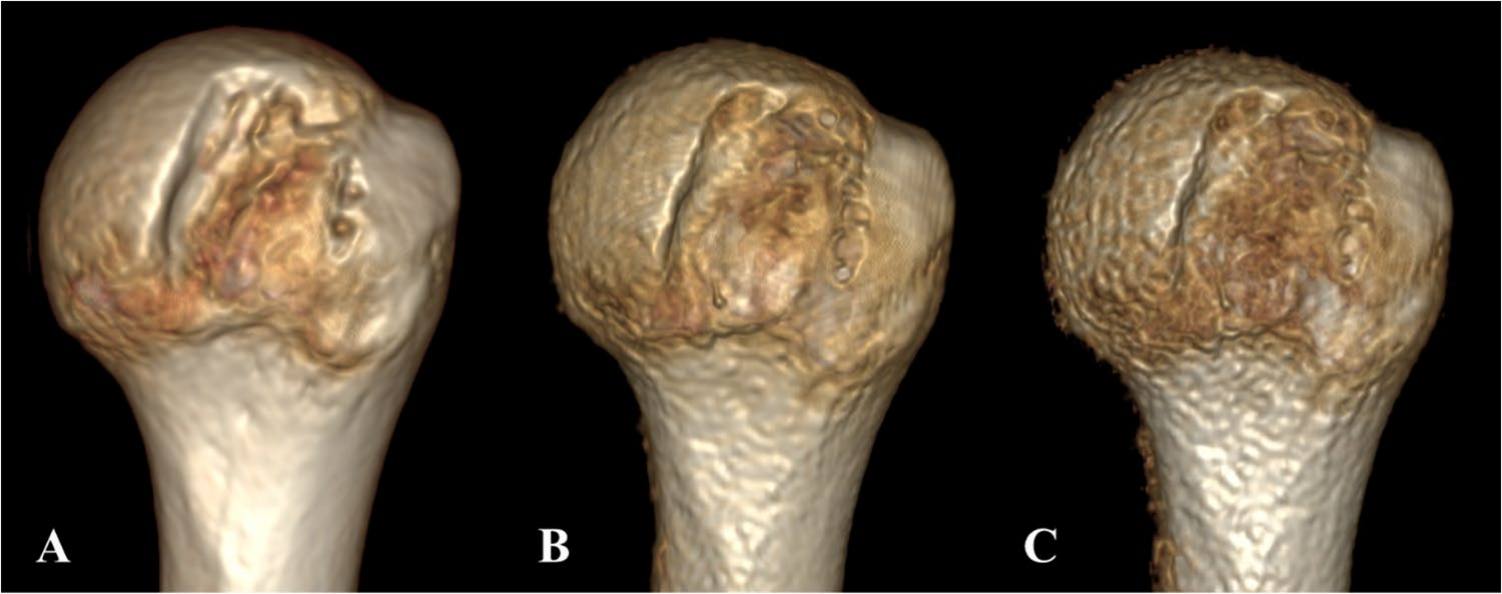
**Comparison of 3D VR of the humerus**. CT (**A**), DL ZTE (**B**), and conventional ZTE (**C**)

All 3D DL ZTE reconstructions from the humerus head yielded excellent VR comparable to gold-standard CT, with a cleaner cortical bone depiction in comparison with the lower quality VR that would be obtained from conventional ZTE as illustrated in Fig. 8.

**Fig. 9:**
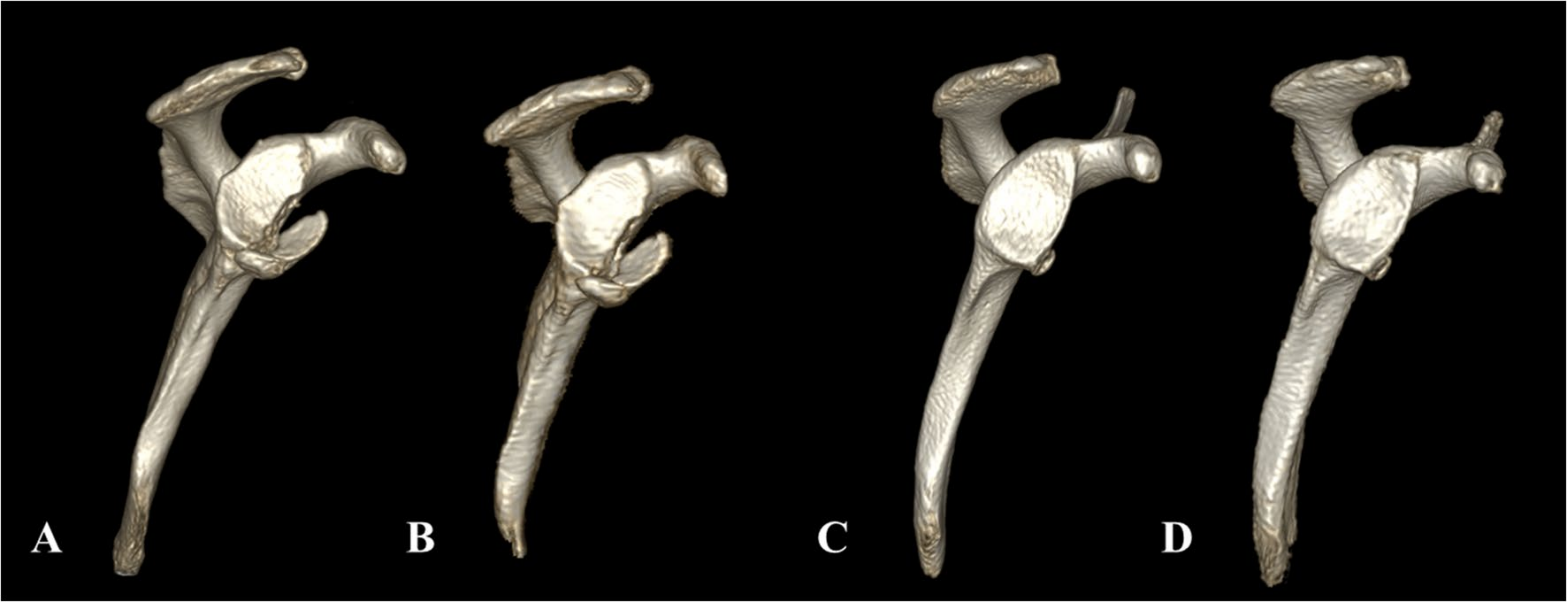
**Comparison of glenoid 3D VR from two patients included in this study**. (**A**, **B**) Large glenoid fracture on CT (**A**) vs DL ZTE (**B**). (**C**, **D**) Small glenoid defect on CT (**C**) vs DL ZTE (**D**)

CT VRs are routinely used to assess bone loss in the pre-surgical setting. In this study, the high-quality 3D volumes produced by DL ZTE showed remarkable similarity to CT renderings, particularly for the humerus (Fig. 8A, B), as well as for glenoid VR in the two selected cases, where the bone fracture and the small defect were comparably depicted on both modalities (Fig. 9).

## Discussion

Our results demonstrate enhanced image quality for DL ZTE versus conventional ZTE, as assessed by the two radiologists (Table 1). DL-enhanced ZTE showed a significant impact on perceived resolution and glenoid lesion conspicuity, increasing the odds of a higher score compared to conventional ZTE (OR = 7.67 and OR = 25.12, respectively; *p* < 0.05), supporting our hypothesis of DL-based ZTE generating improved overall image quality and increasing diagnostic confidence.

Accordingly, DL ZTE provided comparable CT image quality showing high inter-modality agreement (ICCs ≥ 0.986) with a bias of 0.08 mm or less for all bone measurements (GT and HSI).

Although the 3D ZTE acquisition that we propose in this study would be feasible in the clinical setting due to its short acquisition time, a major limitation in its clinical translation is post-processing. The fully automated online processing for CT-like contrast from the ZTE dataset is already available through the oZTEo clinical software (GE HealthCare, Chicago, IL). However, 3D VR is currently still time-consuming and requires background air suppression and manual editing. These renderings must be of high quality otherwise they could potentially miss details of the bones’ complexity and subtle topographic features, which in turn could impact the accuracy of bone measurements [38]. As shown in this study, 3D DL ZTE reconstructions yielded excellent VR similar to gold-standard CT.

The results of this study are consistent with previous studies of ZTE MRI in musculoskeletal applications. Focused on shoulder assessment, substantial inter-modality agreement (ICC > 0.6) between morphological measurements (glenoid morphology and Hill-Sachs lesions) and grades (nominal/ordinal grades for overall injury such as the presence of cysts) from CT and ZTE images has been reported [24]. More specifically on glenoid bone assessment, a recent study compared measurements of the glenoid width between 3D ZTE MRI technique and 3D CT [25]. This work indicated that both techniques offer similar results with high inter-modality and inter-rater agreement (ICCs ≥ 0.94) with bias ranging from 0.3 to 1 mm for all measurements (ex vivo and in vivo) and both readers. In addition, significant agreement of angular measurements for hip morphology has previously been reported between ZTE MRI and CT imaging, with ICCs ranging from 0.618 to 0.904 [28]. The intermodal agreement demonstrated in our work is higher than previously reported values, with lower biases. We believe that the DL reconstruction method further improves diagnostic confidence compared to conventional ZTE.

Shoulder instability is a major concern in current orthopedics and sports medicine practice. The interaction between the multitude of factors at play renders this condition challenging for the surgeon. The emergence of the glenoid track concept for bipolar bone loss assessment has initiated a new area of research, offering promising prospects for advancing our comprehension of glenohumeral instability in the future. Nevertheless, the diversity and lack of consensus within the existing literature, reflecting a range of approaches and imaging techniques for quantifying bone loss, show no clear evidence of reproducibility.

The imaging-based assessment methodology described in this work relies on the 3D acquisition with MPR, which allows for manipulation of the CT/ZTE images to obtain the optimal orientation and plane for evaluating the en face glenoid and the short axis of the Hill-Sachs lesion. Recent studies suggest that using 2D MPR is both easier and more reproducible than relying solely on 3D reconstructions for assessing bipolar bone loss and determining on-/off-track lesions [6, 36, 38].

Having the routine MR exam besides the MR bone imaging gives the additional advantage of showing soft tissue injury in a one-stop imaging setting without ionizing radiation. Due to the inherent soft tissue contrast enhancement, MRI is superior in the identification of the rotator cuff attachment for HSI measurement, as well as providing exquisite conspicuity of the injured capsuloligamentous and labral soft tissue structures, aspects that would otherwise be missed in CT examinations.

In 25 patients (5 females, mean age 35.92 ± 16.44) included in this study, the MR report revealed additional clinical findings that were not observed on the CT scans, such as rotator cuff tears and labral tears. These findings were paramount for preoperative planning and affected the patient management since they could have caused post-surgical failure when ignored.

We acknowledge the age of our group of patients is not consistent with the common age distribution for anterior shoulder instability [4, 5], as we have not excluded any patient by age, including 14 patients older than 40 years. However, the goal of this study was to assess the diagnostic performance of DL ZTE, and the cohort provided the necessary variability. On the other hand, we must note that the quantitative arms of our study only include osseous measurements conducted by a single radiologist during a single assessment. Consequently, we were unable to evaluate interobserver or intraobserver agreements. Based on the literature discussed earlier, we anticipate similar variability as the inter-modality agreement reported here, meaning that differences between measurements from different modalities (by a single rater) would be comparable with differences between measurements performed by different observers (or same observer at different times) on the same modality.

In conclusion, the recent integration of DL-based image reconstruction has further enhanced the quality and efficiency of ZTE imaging, bringing it closer to the gold-standard CT for glenohumeral assessment, with equivalent osseous quantification. DL ZTE has a paramount impact for patient population prone to recurrent dislocations, simplifying the preoperative evaluation combining the ZTE sequence in a routine shoulder MR exam, and minimizing radiation exposure from CT.
